# Horse Shoeing

**Published:** 1877-09-20

**Authors:** 


Horse Shoeing.—This is not a medical topic, and yet medical practitioners, more, perhaps, than any other class, need to know how a horse should be shod.

The length of time a shoe should be worn will, of course, depend upon the kind of work the horse is doing, and the sort of roads over which he travels.

In four to six weeks the hoof will have grown too large for the shoe, which will press inward upon the soft parts of the foot, and the horse will become lame. Before this occurs the shoe should be reset, or a new pair put on. As a general rule a saddle horse will ride better without corks on his shoes. The shoe should be made to fit the foot, and not the foot the shoe. It should rest firmly and uniformly upon the outer rim of the hoof so as to require little or no dubbing off of the hoof by the rasp. Three nails on a side are enough, to be driven in with such inclination as to come out at a point about one and a half inches above, and yet so shallow as not to touch the quick. To know how to do this properly the smith must study the anatomy of the horses foot. A small, tough nail, made to fit tightly the hole in the shoe, should be used, otherwise the shoe will soon become loose.

The frog in the foot may be lightly trimmed, so as to remove any jagged portions, but should not be cut or rasped off, as is usually done. It is somewhat elastic in structure, and is evidently designed to lessen concussion and divide the pressure upon the foot. The hoof should not be burned in fitting the shoe, as is commonly done. Unless your smith is very trustworthy it is well to stand by and see your horse shod. Shoes wear out much sooner if your horse stands on a plank floor, and the horse will also be more likely to become lame.

These suggestions will, perhaps, be regarded as simple and unnecessary by many; but the young practioner, and even older heads, who have not observed closely, will find them profitable. It was not until after long years, during which the writer suffered much loss and inconvenience from lame horses, that he became acquainted with these simple facts.

